# The Challenge of Defining Laterality in Horses: Is It Laterality or Just Asymmetry?

**DOI:** 10.3390/ani15030288

**Published:** 2025-01-21

**Authors:** Kevin K. Haussler, Sarah S. le Jeune, Russell MacKechnie-Guire, Selma N. Latif, Hilary M. Clayton

**Affiliations:** 1 College of Veterinary Medicine, Lincoln Memorial University, Harrogate, TN 37752, USA; 2 Department of Surgical and Radiological Sciences, University of California-Davis, Davis, CA 95616, USA; sslejeune@ucdavis.edu; 3 Equine Department, Hartpury University, Gloucester GL19 3BE, UK; russell.mackechnie-guire@hartpury.ac.uk; 4 Pferdepraxis Vetcheck GmbH, Wierezwil-Rüberi 273, 3255 Rapperswil BE, Bern, Switzerland; selmalatif@vetcheck.ch; 5 Large Animal Clinical Sciences, College of Veterinary Medicine, Michigan State University, East Lansing, MI 48824, USA; claytonh@msu.edu

**Keywords:** symmetry, homologous, hemispheric specialization, motor bias, kinematics, anatomical variants, limb dominance

## Abstract

The defining characteristic of laterality is the dominance of one side of the brain controlling specific functions of paired organs or on one side of the body. Structural and functional asymmetries are ubiquitous in horses and range from anatomical features (e.g., the length of long bones) to the gathering of sensory information (e.g., which eye is used to observe unfamiliar scenes) and motor functions (e.g., left–right differences in locomotion). There is a common tendency to assign observed structural or functional asymmetries to lateralization, which often involves more than a simple left–right difference in observed behavior. This narrative review explores the concept of laterality relative to the structural and functional asymmetries reported in horses. Inconsistent and poorly defined terminology, a widely disparate methodology, and a lack of standardized thresholds make it difficult to assess the presence or degree of laterality. Within this context, there seems to be limited evidence of laterality in horses and much more prevalent and stronger support for structural and functional asymmetries due to a wide range of well-established behavioral, nociceptive, and biomechanical mechanisms. The authors caution against generalizing the idea that all observed structural or functional asymmetries in horses are due to laterality.

## Simple Summary

Humans display handedness in which the right hand is often preferentially used to complete complex tasks, such as writing. Animals often display left–right differences in behaviors, such as the ease of turning in one direction versus the other. These differences are sometimes assumed to be caused by “laterality”, where one side of the brain controls activities on one side of the body. While this concept is well-established in humans, there is limited evidence supporting the idea that the brain’s lateralization is the primary cause of these left–right differences in horses. This study highlights the complexity of interpreting observed asymmetries in horses, as factors like injury, pain, and altered biomechanics may also play important roles. While laterality might be advantageous for some functions, its effects on horses are not always clear. The goal of this narrative review is to offer a clearer framework for understanding laterality and asymmetry in horses, which could improve clinical practices, enhance training methods, and ultimately lead to better care and performance outcomes for horses.

## 1. Introduction

Brain function in animals is lateralized, meaning that the left and right sides of the brain process information differently [1]. The term ‘hemispheric specialization’ describes these lateralized neurophysiological processes within the cerebral hemispheres, which contribute to observed left–right differences in behavioral, sensory, and motor characteristics [2]. Handedness is the most common example of laterality, where 90% of humans are right-handed [3]. The use of one paired limb or body segment over the other is also common in horses, but supporting neurological studies are lacking to confirm that hemispheric specialization is the predetermined dictating factor. When using cerebral lateralization as the sole explanation for laterality, it is assumed that all behavioral, sensory, and motor asymmetries are linked and lateralized to the same or respective side of the body [4]. While most humans are right-handed, lower limb dominance (i.e., footedness) has been reported to be 62% right foot, 8% left foot, and 30% mixed bias [5]. There is strong support for the presence of laterality in animals, but many exceptions and contradictions have been reported on the strength and direction of observed left–right differences [6]. While some individuals exhibit strong laterality for certain attributes, other structural or functional attributes have weak or no significant laterality evident [7].

From an evolutionary or biological perspective, laterality is often judged to be a useful or adaptive process that has helped a species survive [8]. Lateralized behavioral responses in animals are theorized to provide quick flight or avoidance responses, the diversification of brain activity (e.g., monitoring for predators while searching for food), environmental adaptation, and enhanced cognitive skills [7,9]. However, there is little supporting evidence that left-lateralized features are more biologically or clinically advantageous (or determinantal) compared to animals with strong right-lateralized features [7]. While laterality may be advantageous for some functions, it may provide no clear advantage or even be a disadvantage for other functions [8,10]. From a physiological perspective, it is not always clear why laterality is detrimental as a horse may have distinct structural or functional asymmetries, but it is still perfectly able to see, smell, graze, move, and perform. However, from a training and clinical perspective, left–right differences are often judged to be undesirable with a need to be corrected or modified in some manner [11]. Equine welfare is a frequent topic of concern as it relates to the behavioral and other systemic effects of laterality in horses [12,13]. The challenge is determining what specific attributes are most predictive for assessing a horse’s physical and mental health and well-being.

The assessment of laterality is typically based on observations of left–right differences in behavior, visual responses, and locomotor characteristics, but we often do not know the specific cortical process or underlying etiology of the lateralized features. The difficulty of inferring causal mechanisms for observed behaviors lies in the complexity of the neural, biomechanical, and environmental factors that contribute to lateralized features. While behaviors may appear to reflect functional asymmetry, the underlying processes could involve intricate interactions among cortical, subcortical, and peripheral systems, making it challenging to isolate a single causal factor. Additionally, lateralized behaviors may be influenced by adaptive or compensatory mechanisms that obscure their origin. For instance, an observed preference for one side in locomotion or visual responses could result from neural development, environmental shaping, or even pain or injury rather than inherent cortical asymmetry. The multifactorial nature of these behaviors highlights the importance of integrating neuroanatomical, physiological, and ecological data to better understand the etiology of lateralized traits.

In this narrative review, we use horses (*Equus caballus*) as a representative model due to the large body of literature that describes perceived left–right differences during both unridden and ridden activities and the effects of human interaction [14]. The goal is not to provide a comprehensive assessment of all aspects of laterality but to highlight and provide a critical assessment of the underlying assumptions, methodologies, and conclusions that need to be considered (or reconsidered) in discussions about the strength and clinical relevance of measured left–right differences in sensory, behavioral, and locomotor parameters. Our intent is to develop an operational framework to better understand perceived laterality and to assess structural and functional asymmetries in animals. An improved understanding of the types and causes of asymmetry is needed to better determine clinical relevance, improve diagnostic methods, and inform managerial and training practices.

## 2. Laterality

### 2.1. Neurophysiologic Basis

Lateralized brain function is theorized to be the initial driving force for the development of left–right differences in observed sensory, behavior, or motor activities [15]. In humans, language is typically assigned to the left cortex, and visuospatial skills are associated with the right cortex [2]. In animals, the left hemisphere is reported to be associated with more proactive or routine behaviors (e.g., decision making in familiar circumstances and the manipulation of objects); however, the right hemisphere is associated with more reactive responses (e.g., intense emotions, rapid responses in novel settings, and escaping from predators) [16,17]. Hemispheric specialization is also reported to drive special sensory functions such as eye dominance and lateralized auditory and olfactory functions [18,19]. The authors have chosen to use the term hemispheric specialization as it implies a structural or functional reorganization of neural circuitry to meet certain biological needs (Table 1) [8]. Neural structures with measurable left–right anatomical differences may be present within a wide range of vertebrate species [20]. However, the relationship between structural symmetries in neural tissues and lateralized behavior is poorly understood [21].

Different mechanisms have been proposed to explain the presence and development of laterality in animals, which include genetic basis [22], visual development [23], hemispheric specialization [24], and evolutionary selection bias [9]. There are numerous reported claims of the biological significance of laterality in horses [25,26]. The traditional explanation for lateralized brain function is that it helps to avoid the unnecessary duplication of neural circuitry and reduce interference [1]. Laterality is considered advantageous in environments that require simultaneous attention to more than one type of stimulus. Animals with weak cerebral lateralization are reported to be unable to attend to more than one task at a time, and they are more easily stressed than animals with strong lateralization [1]. Horses have developed several unique adaptations to ensure their survival, which may include laterality. The role a specific motor, sensory, or behavioral pattern plays in the overall health and welfare of an individual is unknown. As most studies only measure a single experimental parameter at a single point in time, the cumulative biological and long-term effects of laterality and left–right asymmetries are largely unknown and speculative at best [6]. A limited number of studies have evaluated the longitudinal effects of motor laterality and structural asymmetries (i.e., hoof shape) on measures of performance [27]. Determining which asymmetry variables should be monitored to assess the long-term effects of laterality is complicated and costly due to a long list of confounding variables and known contributing factors [25].

### 2.2. Individual Versus Population Prevalence

Laterality has been reported at both the individual and population levels [28,29]. Laterality at the individual level means that each animal exhibits its own left or right bias (Table 2), while the overall population has a relatively equal left–right distribution [21]. On the other hand, population-based laterality means that most of the individuals within the sample population share the same directional bias [30]. However, most studies do not specify or make the distinction that group behavior only refers to a specific methodology used in the sample population (i.e., internal validity) and may not be applicable to the overall equine population (i.e., lacking external validity) [31]. Conflicting results are often reported when additional studies are conducted, or feral animals are assessed in their natural environments [17,32]. It has been hypothesized that if an individual’s directional bias differs from the surrounding societal network of a herd, flock, or school, then the most advantageous activity during predator–prey interactions is to align with population-based laterality to increase the probability of escape and avoid mass chaos [9,21]. A phylogenetic analysis of laterality across 119 different species revealed that 51% had population-level laterality, 17% displayed individual-level laterality, and 32% had no evidence of laterality during observed behaviors [28]. Therefore, it seems that there is moderate evidence for some measures of laterality across species, but it is largely case-dependent.

### 2.3. Laterality in Horses

**Figure 1:**
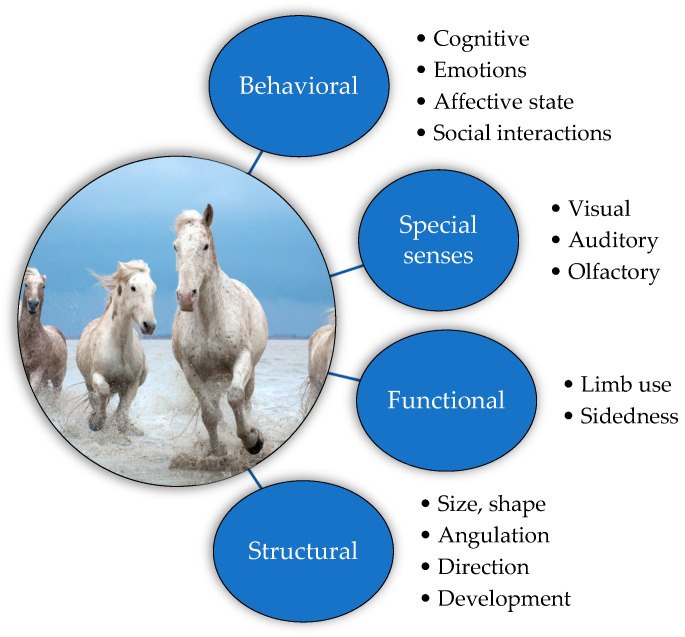
Characteristics that have been attributed to laterality in horses.

Laterality in horses has been characterized by left–right differences in behavioral responses, sensory mechanisms, and motor activities (Figure 1). While a wide range of left–right differences in structural and functional characteristics have been attributed to laterality in horses, there seems to be a lack of understanding or confusion as to what exactly constitutes laterality [13]. The biomechanical and neurophysiologic factors contributing to laterality are likely to change over time [21]. In early development, some lateralized cortical functions are now considered innate physiological processes [4]. However, as an individual accumulates repetitive-use injuries (e.g., osteoarthritis), pain and altered mechanoreception may become primary drivers of observed left–right differences [25]. The challenge is in identifying true laterality (i.e., hemispheric specialization) and differentiating it from structural or functional asymmetries due to other common and well-defined mechanisms [33,34].

A large body of research in the scientific literature addresses specific characteristics of both structural and functional asymmetries in horses without the consistent use of terms and standardized definitions for laterality [13,25]. Descriptions for a neurophysiologic basis of laterality include hemispheric dominance [35] or specialization [8]. Left–right differences in motor function have been described as a preference [25], idiosyncratic [36], or bias [6]. Lateralized truncal and tail movements have been described as directional bias [6], sidedness [37], crookedness [38], and hollowness [39]. The disproportionate use of one limb over the other has been described as limb preference [40] or dominance [41], handedness [42], and leggedness [43]. Finally, the terms morphologic (i.e., structural) and functional asymmetries have been used to describe the anatomic and biomechanical characteristics of laterality in horses [15].

It is apparent that standardized terms and clear definitions are needed to better understand the basic mechanisms for describing and differentiating cerebral laterality from structural and functional musculoskeletal asymmetries. In humans, ambidextrous (‘*right-handed on both sides*’) is a term used to describe the near-equal use of the left and right hands. In the absence of hands, ambidextrous is not an appropriate term to use in horses. Ambilateral or mixed use are alternative terms that have been used to describe the absence of left–right sensory or motor bias in animals [1,12]. The authors have opted to use the term “mixed use” as it applies to horses. The lack of standardization makes it difficult to decide how best to measure asymmetries and evaluate their perceived ecological or clinical relevance. To provide a more standardized and cohesive approach to understanding laterality in animals, the following questions need to be carefully addressed.

- What is laterality?
- What causes laterality?
- Can we measure laterality?
- What are the different types of laterality?
- How does laterality differ from asymmetry?
- What are the relevance or clinical effects of laterality?
- Is laterality helpful or harmful?
- Can and should we change laterality?

## 3. Structural Asymmetries

Symmetry is a commonly observed phenomenon characterized by a uniform radial (e.g., starfish) or bilateral (i.e., left-right) pattern in specific morphological or functional features. Bilateral symmetry occurs when paired body parts are present as mirror images on the left and right of a midline plane. Structural asymmetries are defined as differences in the size, shape, position, or orientation of homologous body parts (Table 3) [44]. Asymmetry is commonly described as a morphological or structural difference between the left and right sides of the body or the absence of symmetry [45].

In horses, structural asymmetries are commonly found in paired left–right segments or body regions [46]. Parameters used to assess contralateral asymmetries include left–right differences in morphology, orientation, proportions, and the relative position of paired structures or sides of the body (Table 4). Horses that have the most difficulty in standing square often have more structural asymmetries compared to horses that naturally stand more evenly [44]. Hemispheric specialization is characterized by lateralized behavior and movement patterns, which, over time, can contribute to left–right differences in muscle development and structural asymmetries [47]. The circumstances under which laterality transitions into structural asymmetry are unknown.

The equine hoof is a dynamic structure, and within paired limbs, the hoof with the lower angle is often subjected to higher loading [50]. However, it is unclear whether higher loading (i.e., function) affects the hoof angle (i.e., structure) or if the hoof angle affects hoof loading [44]. Foals are born with symmetrical fore-hooves [57] but, when grazing on pasture foals, often develop uneven hooves over the first few months of life [48]. Due to limb and neck length discrepancies, foals graze with one forelimb protracted and the other retracted [48]. This grazing posture allows the foal to lower its neck and head sufficiently to reach the ground. Approximately 50% of foals have been reported to protract a specific forelimb while grazing [48]. In this position, loading is concentrated on the heels of the protracted forelimb and on the toe of the retracted forelimb. With the limbs in this asymmetrical position, the gravitational force differentially loads some limb structures [58]. Consequently, the habitually protracted hoof develops a flattened shape with low heels and an acute dorsal hoof wall angle, while the retracted hoof develops a more upright shape with long heels [27]. In feral horses, there is no population bias for placing one forelimb in front of the other during grazing, which suggests that any existing preferences of this type in domestic horses are entrained [17]. Thoroughbred and standardbred racing horses are reported to prefer to graze with the left forelimb in front of the right, whereas Quarter Horses display no bias [31,35]. Therefore, limb preferences while grazing might be influenced by the training and racing direction (e.g., the leading limb during gallop) [6], or diagonal limb pairs while trotting [59] might override any whole-body lateralized motor bias, such as forelimb advancement or hind limb stabilization.

## 4. Functional Asymmetries

Functional symmetry implies a nearly equivalent use or capability (e.g., strength and dexterity) of paired structures (Table 5). From a clinical perspective, functional symmetry is often considered the preferred condition and is used as a criterion for judging gait (i.e., soundness) and ridden performance [36]. Functional asymmetries are characterized by left–right differences in the observed frequency of use (i.e., quantitative) or functional (i.e., qualitative) capabilities of paired body segments [60]. While most paired limbs may not exhibit pronounced structural asymmetries, functional asymmetries are common in horses as one limb is often positioned or observed to be used with greater prevalence during activities [28]. The challenge is determining how much asymmetry is judged to be acceptable (i.e., within a defined reference range) versus judged to be pathologic and require immediate therapeutic intervention.

The proportion of left–right differences in the fore- or hind limb used to initiate movement or avoid obstacles has been used to assess the functional asymmetries associated with laterality [36]. Vertical motion asymmetries of the poll, withers, and pelvis are used routinely to assess gait abnormalities and lameness (Table 6) [61,62]. Similarly, functional asymmetries are used to assess a rider’s skill and to judge equestrian events [52,63]. For example, Quarter Horses used in reining competitions perform movements in both the left and right directions and are judged based on functional symmetry [64].

## 5. Measures of Laterality

### 5.1. Methodology

A wide variety of methods have been developed and described for the qualitative and quantitative assessment of structural and functional asymmetries in horses [25,60,73]. However, there continues to be a general lack of clearly defined criteria and methodology needed to assess laterality in horses [6]. Even with the large body of research evaluating motor bias in humans, clear conventions for defining, testing, or measuring handedness are absent [74]. Studies that use different methods for testing locomotor biases are likely to assess different aspects of motor function (e.g., the limb position at stance versus the leading limb during canter), which would be expected in order to produce different or potentially conflicting results. Using locomotor tests that are comparable to the movement and skills required during work or athletic competition is likely to elicit the most relevant and useful results about locomotor biases [6]. Standing or walking activities are expected to place minimal demands on the neuromuscular system, where either the left or right limb could be readily used. However, more complex or demanding activities require a higher level of focus and musculoskeletal recruitment, where lateralized motor bias might become more evident [16,75].

### 5.2. Research Setting

While structural asymmetries may be fixed within an individual horse (e.g., osseous asymmetry), many functional and behavioral asymmetries may have varying levels of left–right bias that fluctuate depending on the measured attribute or the environmental setting. Studies on laterality are theoretically conducted using normal horses in a neutral setting. However, most laterality studies are conducted in a controlled, experimental setting for the convenience of the human observer [62,65], in contrast to horses being assessed at liberty or in their native environments (i.e., feral horses) [15,17]. Experimental settings are used to control confounding variables and are designed to capture a single type of activity repeatedly within a short period of time. The disadvantages include extensive human interaction [76,77], the use of novel stimuli [78] or experimental settings [79], and the varying levels of training and habituation required to capture the expected response [80]. Different levels of restraint during data collection include the use of a halter and lead rope while standing alone, being held or led by a human handler, being ridden, or allowed to move freely within a confined area [12]. Many of these factors negatively impact the ability to assess laterality in horses. Observing behavior in an uncontrolled or natural setting provides the opportunity to observe spontaneous events with minimal human interference [31]. The disadvantages are that a wide range of behaviors and movement patterns occur naturally, but only a single aspect might be of interest, or the attribute might occur infrequently over the course of the observation.

### 5.3. Outcome Parameters

While many structural and functional asymmetries are readily observed and easy to measure within an individual horse, the ability to identify a specific criterion that can be reliably measured within a sample population and then used to predict laterality is difficult [79]. Well-designed studies consist of distinct measures of left–right differences that provide clear outcomes; however, some observations may be quite subjective and prone to misinterpretation [6,81]. Asymmetry is often judged to be present or absent (i.e., dichotomous) [33] and lateralized to one side of the body (i.e., left and right) without considering that some horses may functionally use both the left and right sides of their body equally (e.g., a mixed response) [15], or that there may be a spectrum of asymmetry (e.g., mild, moderate, or marked) or expression of laterality (e.g., ranging from weak to strong) [82,83].

The response to a visual, auditory, or olfactory stimulus may be recorded as a spontaneous, single event (e.g., sniffing feces) [31]. In other studies, the time required to accomplish a specific task or the duration of the activity is recorded [84,85]. However, some studies have no defined time frames, especially when observing feral horses in a natural setting [15]. Only a few studies have used more objective or physiological parameters to assess laterality, such as heart rate [18], heart rate variability [86], electroencephalography (EEG) [87], or thermal imaging [86]. Even fewer studies include appropriate controls to characterize the relative contribution of confounding variables to observed left–right differences [19].

Locomotor parameters seem to be the most reliable and consistent outcome measures used across studies as they can provide clear, objective measures of left–right differences in limb use [88]. Forelimb bias has been assessed in horses while initiating movement [36], grazing [31], trailer loading [40], and racing [89]. However, these activities require significantly different mental and physical demands, which may contribute to conflicting results in reported sensory and motor biases [6]. Some parameters are highly linked to the activity itself (e.g., the leading limb used while racing through a turn) and are not associated with laterality itself [89]. Training sessions are often required in experimental settings, where 10–20 repetitions are typically needed to achieve a desired training response before recording data [12,84]. The number of recording sessions has a significant effect on observed laterality, where measures of laterality decrease over time due to habituation, training effects, or a reduction in the novelty of the stimulus [12,40]. Caution is warranted as the assumed difficulty of the task is often based on the perception of the human examiner rather than the mental or physical attributes of the individual horse [16].

### 5.4. Human Influence

Another important consideration is the inappropriate transference of human emotions and motivations to horses and other animals (i.e., anthropomorphism) [90]. As external observers, we can only perceive what animals do (often in artificial environments) and assign our human perceptions to those activities. Human bias has a significant impact on the design, implementation, and interpretation of observed behaviors or movement patterns in equine subjects [33].

Being aware of the effects of observer bias, anthropomorphism, and researcher perspectives is an important attribute to consider when assessing the outcomes of laterality studies in horses. Observer bias can significantly influence the outcomes of laterality studies, particularly when subjective parameters are used. Human observers may unintentionally project their expectations based on previous experiences and theoretical frameworks, leading to systematic errors in study designs, data collection, and interpretation [17]. Observers with preconceived notions about equine laterality may be more likely to attribute lateralized behaviors to specific populations (e.g., feral horses) [15], biological mechanisms (e.g., social interactions) [91], or horse–rider interactions (e.g., rein tension) [33]. This highlights the need for blind and standardized protocols to minimize such biases.

Researchers may also subconsciously apply human-centric frameworks of handedness to equine behavior, leading to oversimplifications or misinterpretations of asymmetries. While human laterality is predominantly limb-specific and task-dependent, equine laterality may be influenced by many different ecological and biomechanical factors [92]. Researcher perspectives, shaped by scientific training (e.g., behavior versus biomechanics) or athletic disciplines (e.g., show jumping versus dressage), can also impact the framing and interpretation of laterality studies [21].

### 5.5. Interpretation

Most laterality studies evaluate a single parameter or individual trait (e.g., dominant eye, leading limb) and then make broad conclusions about the presence and importance of laterality [84,93]. However, when multiple laterality tests are included within a study, the results are often inconclusive, with little to no correlation between multiple combined tests [12,33,94]. Most laterality studies have been conducted with the objective of linking combined traits to either the left or right side of the body (e.g., linking the left side of the brain to right-sided motor functions) [17,40]. Horses that initiate movement with the right forelimb are described as having more favorable responses when approaching an ambiguous box [12,40]. A shortcoming in this approach is that there is an assumption that there is a one-to-one relationship between the selected parameters, which excludes or ignores other important contributing factors, such as fear, pain, training, and human interactions [11,35,77]. In a study assessing forelimb advancement during grazing and the concurrent unweighting of a hind limb, there was no ipsilateral motor bias in these relatively easy-to-assess locomotor parameters [31]. Other locomotor mechanisms, like the use of diagonal limb pairs, may override any laterality bias [95]. In addition, left–right differences may be quite pronounced (i.e., highly lateralized) for one specific parameter (e.g., limb advancement), but that same degree of laterality might not be evident or could even possibly be reversed for another sensory, behavioral, or locomotor parameter within the same individual [31].

### 5.6. Statistical Analysis

There is a clear bias in the literature on laterality where dichotomous outcome variables (left or right) are measured and used to draw conclusions about the proportion of left–right differences [33]. A common method of assessing left–right differences is the laterality index (LI), where LI = (Left − Right)/(Left + Right) [18]. A laterality index value of 1 indicates an exclusive left bias; values close to −1 indicate a right bias; and values near 0 indicate no left–right bias. A laterality index value close to 0 has been used in many studies to indicate no significant left–right difference [6]; however, the laterality index does not include horses that use both limbs or sides of their body in similar proportions (i.e., mixed bias). A more accurate and useful laterality index would, therefore, include all possible outcomes—left, right, and mixed-use.

Some studies are designed so that only left–right responses are produced [96], while others are designed so that left–right and mixed-biased results are assessed [12,15]. A mixed-use bias is often disregarded [6] or not discussed in laterality studies as the objective is to only assess left–right differences. For example, a study that assessed forelimb advancement during grazing reported that the left forelimb was advanced 41% of the time, the right forelimb was advanced 9% of the time, and a mixed response was noted in 50% of horses [31]. The authors concluded that the horses displayed a statistically significant left forelimb bias when, in fact, most horses had no laterality or motor bias. Similarly, another study concluded that horses had a left forelimb bias (i.e., 8% left; 2% right) when 90% of horses had mixed limb use [33]. All possible combinations of left, right, and mixed biases need to be considered if we are to fully understand laterality in horses. The threshold for detecting left–right differences at a population level has been reported to be about 35%, with the minority type making up 10 to 35% of the population [30]. However, this threshold assumes that the other 65–90% is strongly lateralized and that there is no mixed bias (i.e., both left and right use equally) present.

### 5.7. Clinical Relevance

While a parameter may demonstrate a statistically significant left–right difference (*p* < 0.05), the relative biological differences (e.g., age, sex, breed) and clinical relevance are often speculative [97]. If extreme, structural and functional asymmetries are known to be detrimental to health, performance, and welfare [50]. Many factors are known to influence measures of laterality; however, the specific impact of each variable on a measured response (e.g., the effect of the presence or absence of human contact) has not been demonstrated in randomized, controlled clinical trials. There is a critical need for some level of consensus in study design, the analysis of data, and conclusions drawn if laterality is to be considered a viable biological adaptation and clinically relevant issue in horses [98]. Appropriate clinical thresholds or standardized reference ranges for establishing the transition from a physiological condition (i.e., laterality) to a pathological or diseased state (e.g., lameness) are largely unknown [69,70,71]. The level of judged clinical significance of any left–right difference likely varies substantially between examiners [99].

## 6. Contributing Factors

Locomotor asymmetries in horses are known to develop along a spectrum of increasing severity and may be a consequence of hemispheric specialization (i.e., innate), congenital factors (e.g., genetics), developmental factors (e.g., suckling or grazing postures), acquired factors (e.g., injury or disease), learned responses and training effects, human interactions, the testing methods used, and environmental factors (e.g., feral). The challenge is trying to determine the relative contributions of each of these factors to the overall observed behavior or isolated locomotor response. Genetic factors have been implicated in the development of laterality in horses; however, strong foundational research to support a genetic basis is lacking [36,100]. It is likely that left–right differences are produced by several overlapping mechanisms and not by a single inciting cause. Therefore, attributing all observed left–right differences in equine behavior, including sensory functions and locomotion, to hemispheric specialization fails to capture the full complexity and interwoven interactions of these biological mechanisms.

### 6.1. Demographics

There are many reports of age, sex, and breed differences in motor function assigned to laterality [17,35]. Laterality due to hemispheric specialization is considered an innate, evolutionary-derived physiological process [101]; however, it has not been well established that horses possess innate laterality. It is likely that some laterality characteristics are present at birth [59], but most studies on foals report a high prevalence of mixed-use bias. Examples of behavioral and motor bias in foals include the side chosen when suckling (65% mixed bias) [102], forelimb advancement while grazing (54% mixed bias) [48], and derailment at a trot (69% mixed bias) [79]. The effects of a mare’s laterality on measures of laterality in the foal are unknown. Some studies suggest that movement asymmetries develop with age [31,103], while other studies report no association with age [104]. Most measures of laterality have been performed in young or adult domestic horses where there are significant human influences on handling and training [25,33], which may override the effects of any innate hemispheric specialization [73].

Sex differences have been reported in laterality studies where females display significantly more right-lateralized responses, and males exhibit more left-lateralized responses [36]. When using the forelimb that initiates movement, obstacle avoidance, and rolling direction, differences were found between males and females; however, no differences were found when the gallop lead was used as a test of locomotor bias [89].

Breed differences have been reported using grazing stance as the bias indicator, where a left bias was noted in Thoroughbreds and Standardbreds, but no limb bias was found in Quarter Horses [35]. However, no breed differences were noted in racehorses using the leading limb at a gallop as the bias indicator [89]. Limb preferences can be entrained or generated by breed-specific selection [17,32]. Many breed differences have been reported across numerous physiological parameters in horses; however, a biological explanation for why laterality should be the same or different across breeds is missing [73,103]. Just because a left–right difference can be measured does not always make it meaningful. Many observed breed differences are much more likely due to genetic factors, use, and training (e.g., Standardbred trotter versus pacer) rather than strictly related to hemispheric specialization [103].

Different aspects of laterality have been reported across equestrian activities and athletic disciplines. Horseracing is typically characterized by racing on an oval track in a specific direction, which often requires changes in the leading fore- and hind limbs as the horse navigates turns and straightaways [35,89]. When jumping, horses have been reported to lead with the right forelimb; however, the direction in which obstacles are approached likely affects the leading limb [36]. Trotters tend to be handled and driven on both sides, whereas most other horses are predominantly handled from their left side. The multifidi musculature cross-sectional area has been reported to be significantly larger on the left than the right side in a group of training horses both before and after a course of core training exercises [105]. The reason for this is unknown but could be related to habitually handling or mounting from the same side.

### 6.2. Developmental Factors

It is often difficult to know if laterality is due to innate left–right differences in cortical function or due to training effects, human interaction, or the accumulation of repetitive use injuries [25,77,79]. The neurologic processes and contributing factors to gait asymmetries are likely to change over time. In early development, there may be cortical functions that are lateralized, which would predispose the use of one limb over the other, causing subtle structural or functional asymmetries [48]. However, as individuals accumulate repetitive use injuries (e.g., osteoarthritis), pain or altered mechanoreception often become primary drivers of observed left–right differences, which we characterize as functional (e.g., lameness) or structural (e.g., bone length) asymmetries [46,79]. Conformational defects often induce structural asymmetries such as angular limb deformities, altered joint angles, and differences in limb segment lengths [46,106]. While some of these conformational defects may be corrected or minimized in the young foal, many of these asymmetries persist as functional left–right differences into adulthood [107]. In National Hunt racehorses, 25% of the variables measured exhibited significant anatomical asymmetries [108], which has been reported as a sign of laterality in many other studies.

### 6.3. Acquired Factors

It is often difficult to identify and differentiate the many different etiologic factors that contribute to the development of structural or functional asymmetries in horses [44,108]. All studies on laterality have been performed on presumably healthy animals or horses judged to be sound; however, pain and tissue injuries are common and known to produce lateralized clinical signs [62,68], which may or may not align with the preexisting hemispheric specialization. If an observed left–right difference in locomotor activity has a readily apparent etiology (e.g., hoof imbalance or pelvic fracture), then the difference is typically judged to be due to a disease process and not laterality. Structural asymmetries have been negatively correlated with performance in Standardbred trotters (pelvis) [55], Thoroughbred racehorses (head, forelimb) [109], and horses used in events (head, forelimb, hind limb) [110]. However, the overall prevalence and clinical relevance of structural asymmetries cannot be explained by a few anatomical features but must be considered within the context of complex biomechanical and neurophysiological interactions within the individual horse [108,111]. It is unknown when these acquired asymmetries may override and cancel out or conversely accentuate existing laterality mechanisms [112].

### 6.4. Structural–Functional Interactions

There is often a close interaction between structural and functional asymmetries. Structural asymmetries can and often do induce associated functional asymmetries [50]. Uneven feet and angular limb deformities in young horses are known to induce the asymmetric loading of the joint surfaces and adjacent soft tissues [66,113]. If these developmental defects are not corrected at an early stage, then longer-term functional deficits (e.g., altered tissue loading) are likely, with an increased risk of injury and the development of chronic disease processes (e.g., osteoarthritis). Conversely, functional asymmetries can also induce structural asymmetries where training more consistently in one direction may contribute to the development of structural asymmetries or lameness [46]. In humans, participation in sports that require lateral dominance (e.g., tennis) is often associated with asymmetrical adaptations in bone and muscle [114,115]. Competitive Thoroughbred or Standardbred racing horses on an oval track undergo repeated bouts of running at high speeds in a single direction with alternating straight lines and curves [116]. This functionally asymmetric athletic activity produces an unequal distribution of limb forces associated with galloping on a turn [117]. Functional asymmetries in racehorses are hypothesized to contribute to the development of overuse injuries (e.g., stress fracture disease), catastrophic musculoskeletal fatalities, and over time, can lead to structural asymmetries of the third metacarpal bone [118], femur [119], and pelvis [55,120]. Many functional asymmetries, if severe or used for a long enough duration, produce compensatory movement patterns and structural asymmetries that contribute additional functional asymmetries [65,66]. Therefore, these structural and functional asymmetries are not considered to be laterality under the strict definition of hemispheric specialization [33].

**Figure 2:**
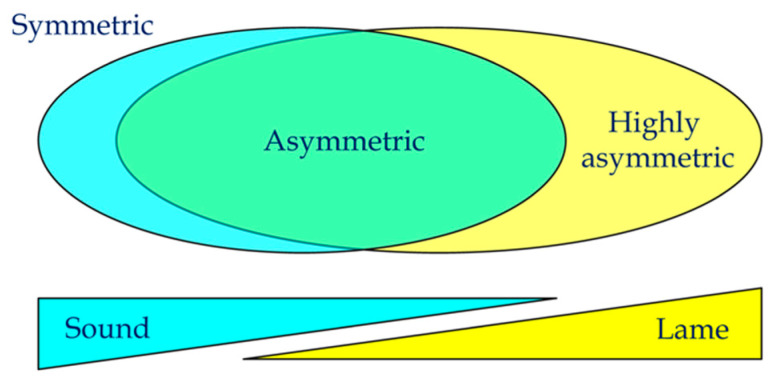
Illustration of the relationship between symmetric and asymmetric gait patterns, which are judged to reflect soundness or progressively increasing grades of lameness.

If we are to minimize the detrimental effects of observed structural and functional asymmetries, then the ability to identify the inciting cause or characterize the pathogenesis of left–right differences is required. In addition, the point when subtle asymmetries or altered gait patterns that are not considered to be clinically significant transition into being clinically relevant in terms of adversely impacting performance is largely unknown and highly variable [68,69]. While a left-to-right difference of 5° in plantar solar angles might not produce overt clinical signs in one horse, it might be the primary source of altered gait or lameness in another [121]. The increased use and reliance on inertial sensors and artificial intelligence to assess gait parameters has made the discrimination between what constitutes a clinically relevant (i.e., pathological) condition that is judged to require medical or surgical intervention from a subclinical or physiologic asymmetry much more indistinct [122] (Figure 2). Horses that are judged to be clinically sound commonly have concurrent structural asymmetries (e.g., tuber sacrale height differences) and asymmetric ground reaction forces, which may not be appreciated during a visual examination [34,123]. Additionally, left–right differences in forelimb hoof shape and angles have been linked to asymmetric forelimb ground reaction forces without an obvious source of pain [124].

### 6.5. Physiological Mechanisms

**Figure 3:**
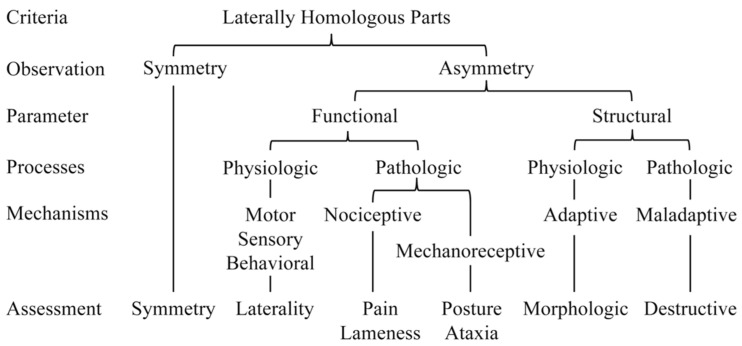
Algorithm for assessing structural versus functional asymmetries and physiological versus pathological processes that contribute to observed left–right differences in horses.

There is a spectrum of physiological and pathological processes that contribute to observed left–right differences in horses (Figure 3). Many of the proposed mechanisms for observed functional asymmetries are considered physiological (e.g., genetic or developmental) and may be defined as laterality [100]; however, pathologic mechanisms and the interaction or transition from a physiological (i.e., adaptive mechanism) to a pathological (i.e., maladaptive) process must also be considered. Musculoskeletal pain and neurologic deficits often induce asymmetric stance and locomotion parameters [25]. From a clinical perspective, it is important to distinguish between the physiological and pathological causes of locomotor asymmetry [37,125]. Defined thresholds and reference ranges are needed to determine when structural or functional differences are judged to be clinically relevant.

Responses to injury can be categorized as adaptive or maladaptive processes. Adaptive processes include an acute inflammatory process, tissue healing and remodeling, exercise-induced muscle hypertrophy, and bone modeling due to increased mechanical loading. Some of these responses may be self-limiting and short-lived, while others may persist throughout the horse’s life and contribute significantly to observed left–right differences in structural and functional attributes [59,89]. Maladaptive processes include chronic pain syndromes, weight-bearing limb laminitis, subchondral bone sclerosis, and fibrosis. While some of these detrimental changes affect bilateral structures, often one side or limb is more severely affected, which may complicate a diagnosis of laterality.

### 6.6. Pathological Mechanisms

Asymmetric movement patterns due to injury or disease are common in clinical practice. Signs of altered weight bearing, including shortened stride lengths and a reduced joint range of motion, are often indicative of left–right differences in limb use [126]. Lateralized motor behavior leads to increased unevenness in the front feet and asymmetry in athletic performance [27]. Structural and functional asymmetries often create increased loading in some tissues and reduced loading in others [66]. Highly loaded structures are often at increased risk for repetitive use injuries and failure [41]. While most musculoskeletal injuries are resolved, some disease processes progress to end-stage disease (e.g., eburnation, ankylosis, and fibrosis). If these disease processes are localized to a single limb or side of the body, then obvious left–right differences in structural and functional features are readily identified [39]. However, chronic pain and asymmetric movement patterns are often associated with cortical changes due to neuroplasticity mechanisms [127,128]. The following question then arises: are these maladaptive neural processes then considered hemispheric specialization?

### 6.7. Nociceptive Mechanisms

The basis of any lameness evaluation is the identification of asymmetric gait. From a musculoskeletal perspective, laterality may be observed in the preferred use of a body part or limb (e.g., leading limb) but must be distinguished from nociceptive stimulation or mechanical restrictions that produce reduced weight bearing in one or more limbs (Table 7) [68]. In lame horses, kinetic and kinematic asymmetries are associated with pain, neurological dysfunction, and restricted movement [39]. The challenge in identifying true laterality or physiological asymmetry is differentiating the observed left–right differences due to pathologic asymmetries (e.g., lameness and ataxia).

### 6.8. Mechanoreceptive Mechanisms

Altered mechanoreception has been described as “micro-klutziness”, which is considered a significant contributing factor to sensorimotor integration and the development of musculoskeletal disease and osteoarthritis in humans [129]. It could be argued that peripheral mechanoreceptors (e.g., muscle spindles and Golgi tendon organs) are just as important in the development of motor biases as hemispheric specialization. Ataxia is defined as the lack of coordination between voluntary movements. Spinal cord compression is a common cause of ataxia and pelvic limb weakness in horses [130]. Abnormal mechanoreception and spinal cord compression produce altered body awareness and compensatory gait patterns that may be perceived as laterality in some horses but are often characterized as a pathological condition.

## 7. Behavioral Asymmetries

Behavioral laterality includes the cortical processes of emotions, affective states, cognition, and social interactions [77]. The series of events theorized to be involved in these behavioral attributes include progression from (1) environmental stimuli, (2) special sensory signaling, (3) afferent hemispheric activation, (4) appraisal (i.e., reward and punishment), (5) emotive response, (6) efferent hemispheric activation, (7) lateralized motor behavior, and finally, (8) observed left–right differences (i.e., laterality) [131,132]. Researchers have used this algorithm to reverse-engineer or predict emotional states and cognition based on observed behaviors (e.g., defecation, head tossing, licking and chewing, pawing, and tail movements) [40]. However, there are many other factors that produce left–right differences in behaviors or locomotor activities other than the emotional state of an animal [12]. The human examiners’ level of experience in dealing with horses and their familiarity with observing behavioral cues are known to have a significant impact on measures of laterality [133,134,135].

### 7.1. Cerebral Lateralization

The lateralization of emotional responses in animals is evidenced by left–right differences in hemispheric activation, where the right hemisphere predominantly controls withdrawal behaviors and responses to threats, escape mechanisms, and intense or negative emotions [4,136]. Conversely, the left hemisphere is reported to be involved in more analytical processes of categorizing stimuli, which is evidenced by approach, familiar or routine situations, and positive emotions. Behavioral responses are often difficult to assess in non-verbal species [137]; however, experimental studies have been developed to capture some aspects of lateralized behavior in horses (Table 8) [12,17]. Ethograms have recently been used to characterize different types of behavior or activity in stall-confined [90] and ridden horses [138]. While many of these observations display left–right differences in body segment use or position, ethograms have not yet been designed to specifically assess the presence or degree of lateralized behaviors.

### 7.2. Emotions

Left–right differences in emotional processing and affective states have been widely reported in animals [4]. Emotions are defined as short-lived moods (e.g., startle response), whereas affective states represent long-term moods (e.g., anxiety) that reflect an accumulation of life experiences (Table 9) [131,142]. Attentional state and emotional reactivity are reported to be elevated when perceived from the left side (i.e., right hemispheric processing) [17]. However, the study of emotions in animals is difficult due to the inability to identify unconscious or subjective experiences (e.g., feelings), which are typically assessed by linguistic reports in humans [88]. Therefore, changes in body position or movement patterns are used as possible indicators of underlying mental or emotional status. Hormone concentrations, body temperature, ear positions, facial expressions, posture, and behaviors, such as approach and avoidance behaviors, can provide valuable information about emotional states in horses [132]. Emotional laterality has been assessed by physiological responses to novel stimuli (e.g., heart rate) [143] and by lateralized motor behaviors such as moving toward or away from a stimulus [60,88,103].

### 7.3. Affective State

Affective states are assessed by measures of arousal (i.e., emotional intensity) and valence (i.e., positive or negative experience) [144]. Arousal indicates the level of engagement or response to a stimulus [131]. For example, fear induces an intense physiological response, whereas boredom does not produce the same physiological changes. High values of valence are characterized by pleasant situations, and low-valence values are attributed to unpleasant situations (e.g., stress, anxiety) [144]. Therefore, positive-valence, high-arousal states represent the activation of a reward acquisition system (e.g., approach), whereas negative-valence, high-arousal states represent the activation of a punishment-avoidance system (e.g., escape) [131]. Affective states can then be assessed in terms of reward and punishment. Previous experience with horses and the observer’s emotion recognition ability both affect the interpretation of a horse’s affective state [133]. However, there is often a poor correlation between a rider’s perception of their horse’s laterality and measures of left–right asymmetries [33,125].

### 7.4. Coping

Affective states in horses have been evaluated via responses to potentially frightening objects (e.g., blue tarpaulin) [86]. Many coping mechanisms in horses are displayed along a continuum from proactive to reactive responses [86]. Escape mechanisms are often characterized by a directional bias [17,91]. Proactive responses may include moving away from or eliminating the source of stress. Reactive strategies include freeze responses, emotional blunting, and unresponsiveness [145]. Horses judged to be proactive show similar stress responses as more reactive individuals, which suggests that behavior may not provide a reliable indicator of a horse’s ability to tolerate a stressful procedure [86].

### 7.5. Cognition

Cognition involves the mental processes of gaining knowledge and understanding and then making decisions based on those experiences. Cognitive processes include learning and the execution of skilled motor activities [140]. Emotions and cognition are closely related, where emotional status can directly impact conscious choices and observed behavioral responses [88]. Other cognitive parameters include attention, memory, judgment, and perception. Different types of work or athletic activities require different levels of attention and skills, which affect laterality [73]. Complex tasks are expected to produce more functional asymmetries due to increased mental and physical demands [7,16].

### 7.6. Social Interactions

Affiliative interactions relate to the desire to form social and emotional bonds with others. As a horse approaches another horse or group of horses, the approached horse(s) may stand still or move toward (i.e., affiliative behavior) or away (i.e., non-affiliative or agonistic behavior) from the approaching horse. Affiliative interactions typically involve a pair of horses standing near each other while allogrooming, swishing flies for each other, and standing in close proximity while grazing or resting [10,91]. Non-affiliative interactions include approaches with the ears pinned back and the head and neck extended, threatening to bite or kick, and chasing [146]. Left–right differences are noted in the direction of approach and type of physical interaction, which is reported to be an indication of hemispheric specialization in both domestic and feral horses. Head-on approaches may occur in agonistic encounters and are typically excluded from affiliative laterality analysis [91].

Mare–foal interactions are often lateralized [141], as are the side of approach and contact by conspecifics [91]. The responses to vocalization from familiar versus unfamiliar conspecifics [19] and responses to human interaction [147] have also been used to assess the lateralization of social behaviors in horses. It has been proposed that the strength of laterality is of greater significance than the direction of movement [83]. In studies that assess multiple laterality parameters within a single population, it is common to find mixed and conflicting results [12,33]. In a group of feral horses assessed for whole-body asymmetries, a right-sided bias occurred for allogrooming, a mixed bias occurred for affiliative approaches, and a left-side bias occurred for the duration of lateral recumbency [10]. It is difficult to ascribe these left–right differences solely to hemispheric specialization when other factors such as environment, training effects, and human interactions likely override many laterality attributes.

## 8. Locomotor Asymmetries

Motor laterality is defined as left–right differences in the use or movement of paired limbs or body segments and is a frequently observed characteristic in horses [45]. Motor laterality has been assessed during standing postures, the initiation of movement, limb use, direction of travel, and canter leads (Table 10) [25].

The general premise stated across laterality studies is that hemispheric specialization is the sole or primary source for observed left–right differences. To substantiate this theory, numerous studies have attempted to link sensory information (i.e., vision and hearing) received on the left side of the body to behavioral and motor responses localized in the right hemisphere and vice versa [4]. This then assumes that all sensory, behavioral, and locomotor attributes will be lateralized in predictable patterns across all individuals and populations studied; however, this is often not the case. While a single measured parameter (e.g., forelimb advancement) may follow this pattern, when several sensory or motor responses are recorded within an individual or sample population, there are often significant inconsistencies in the direction and strength of the lateralization of the observed parameters [83]. Between studies, there are numerous left–right discrepancies in lateralized behavior that are often attributed to the study design, experimental setting, and human interactions [6]. As an example, a left forelimb bias has been reported while grazing [31], but a right forelimb bias occurs in racehorses [89].

### 8.1. Appendicular Laterality

The most common example of motor laterality in horses is the observation of standing or grazing postures where a single forelimb is advanced [31]. Quadrupeds can display a wide variety of stance positions, patterns of limb use, and different amounts of weight bearing across their four limbs [150]. While forelimb observations are commonly used to determine motor laterality, the hind limb is reported to initiate movement in most cases [151]. Postural constraints play a significant role in laterality as the paired fore- and hind limbs must work together to make subtle changes in the center of mass position and produce coordinated limb movements during stance and while traveling at different gaits [89]. A prerequisite to movement is the need to provide a solid foundation for the weight-bearing limbs to support the isolated unweighting and advancement of one or more of the other limbs while traveling in straight lines, turns, or circles [16]. It is not clear which features of motor biases (e.g., hemispheric dominance, limb unloading, or limb retraction) are assessed when the advanced forelimb during grazing is used as the sole indicator of laterality. The advanced forelimb is judged to be the dominant limb (e.g., moved first or used more); however, the retracted forelimb serves as the stabilizing limb and, therefore, bears more weight [6]. Similarly, the hind limbs support the caudal half of the body, often with a protracted and retracted hind limb posture. Therefore, we propose using the terms ‘mobilizing’ and ‘stabilizing’ to describe differences in limb use and function (Table 11). The primary mobilizing forelimb is considered the leading limb and is used to change the direction of travel, whereas the primary mobilizing hind limb is used to establish stride length and velocity [152,153]. Stabilizing limbs preferentially support the body and generate higher ground reaction forces.

Statically, the forelimb that is more frequently protracted during stance or grazing is often judged to be the lame limb [126]. Dynamically, the first limb used to step forward or the leading limb during cantering or galloping is identified as the dominant limb [25]. In humans, the dominant hand is often extended out in front of a person’s body to initiate or perform a task, and the frequency or ease of use of an appendage (e.g., hand or foot) is termed handedness or footedness. The term ‘handedness’ can only be strictly applied to humans and non-human primates since, anatomically, they are the only species with hands. Handedness also implies that there are left–right differences in fine motor skills, such as when writing or using tools [154]. Similarly, footedness implies that one foot is more dexterous than the other [155]. Since horses have a single digit and no opposable thumbs, handedness, and footedness are judged to be unsuitable terms to describe a motor bias observed within the distal limb or hoof.

The term preference (or preferred limb) has been used to describe functional asymmetries associated with laterality [25,28]. Preference implies that a conscious decision is made to use one side of the body or to select one limb to complete a task. As external observers, humans can only make suppositions about the underlying mechanism or motivating factors for functional asymmetries in non-verbal species. Personal preferences in humans are known to readily change with the time of day (e.g., morning person), weather (e.g., carrying an umbrella or not), traffic patterns (e.g., choosing alternate routes), and personalities (e.g., introversion and extroversion) [156]. Since laterality is considered an unconscious or innate process that does not require a conscious decision, preference is judged to be an inappropriate term to describe motor laterality in animals.

### 8.2. Truncal Asymmetries

Truncal or whole-body laterality at liberty (Table 12) has been assessed using behavioral responses [91], mare–foal interactions [141], and the direction of recumbency [10] and rolling [36]. Mare and foal interactions are often assessed for lateralized behavior while lying down and during suckling or locomotion. In humans, infants are often held on the left rather than the right side of the body, which may reflect left stabilizing and right mobilizing or a dominant upper limb [157]. The laterality of mare–foal interactions has been studied from the perspective of the foal approaching the mare, the mare approaching the foal, and their relative positions while standing or moving. A left bias has been reported for foals while standing facing forward on the right side of the mare and while suckling on the left side of the mare [141]. The question then arises as to whether laterality is determined by the foal’s position with its left side in contact with the mare while both standing and suckling (i.e., left laterality) or by the mare’s position relative to the foal (i.e., standing with the foal on the right side versus suckling with the foal on the left side).

Sidedness refers to asymmetries in whole-body movements with an inherent use of one side of the body or ease of movement toward one side during locomotion or equestrian events (Table 13) [25]. Sidedness can be assessed based on visual observations, rider perceptions, learned behaviors, skills, or physical use. The terms straightness and suppleness are used in the dressage training scale [159]. Straightness refers to the alignment of the horse’s axial body segments and the tracking of the haunches relative to the forehand [39]. When the curvature of the axial skeleton is sufficient for the hind limbs to follow the same track as the forelimbs when traveling in either a straight line or on a curve, the horse is said to be straight. The ability to slightly modify spinal curvatures during locomotion is an integral part of the horse’s ability to maintain the verticality of the limbs and trunk when turning rather than leaning into the turn [160].

Suppleness refers to the overall flexibility of the axial skeleton where the ease of and amount of movement is equal to the left and ride sides. Left–right differences often occur where one side of the body is stiffer and less supple [95]. When there is a noticeable left–right discrepancy, the horse is said to have a supple side to which lateral bending is easier or of greater magnitude and a stiff side that is relatively inflexible. However, if a horse is described as stiff to the left, this may imply a reluctance to stretch or lengthen the musculoskeletal tissues on the right side of the body; therefore, both sides of the axial skeleton need to be considered from a whole-horse perspective [11]. Overall, trainers describe 60–80% of horses as being stiff on the left side and more supple on the right side (Table 14). However, there is a large variation in rider’s assessments of rideability for individual horses [161]. Since whole-body movements require all four limbs and both sides of the head, neck, and trunk to work in unison, the issue of sole left or right hemispheric specialization becomes more difficult to justify.

There are no established criteria to define the presence and relative contributions of limb bias versus sidedness within an individual horse [39]. Sidedness and left–right differences in mobilizing and stabilizing limbs are likely significant factors influencing a horse’s ability to perform tasks symmetrically [95]. Riders and coaches often report that horses perform tasks with greater ease and athletic ability when ridden in one direction compared to the other (i.e., directional bias). Biomechanically, a stiffer side may contribute to increased truncal stability, and a more supple side promotes truncal mobility [95]. A combination of both dynamic stability and suppleness is a desirable characteristic for athletic performance [25]. Large differences in spinal kinematics occur between unridden and ridden horses, which makes generalizations about sidedness and laterality difficult [39]. Asymmetries measured within the horse may increase with the presence of a rider [150]. However, no clear associations have been reported between riders’ perceptions of their horses’ sidedness and kinematic asymmetries [37,39].

## 9. Human Influences

By convention, most horses are approached, handled, and mounted primarily from their left side, which likely has a significant impact on the motor bias observed in horses while engaging in both unridden or ridden activities [77]. The effects of a person’s approach style, speed of approach, and direction of gaze have also been reported to contribute to observed differences in the behavioral responses of horses [162]. However, it is difficult to determine the relative contributions of each of these parameters on measures of laterality. It is expected that with increased human interactions and training, horses become more habituated to lateralized handling and stimuli [25,33].

### 9.1. Training Effects

Many riding disciplines require a complex array of movements, many of which require extensive training and repetitive practice sessions. Athletic development requires physiological adaptations to increase the intensity and duration of exercise and training in sports-specific tasks. Symmetrical movement patterns are judged to be important for most equestrian activities (e.g., dressage and showjumping). It is common practice to work horses equally to the left and right during training and riding with the goal of supporting the development of symmetrical strength and movement patterns. However, there is little evidence to support the effectiveness of this type of work in reducing motor biases in horses [6]. Similarly, it is unknown if horses with lateralized movement patterns should be preferentially selected for enrollment in specific sports activities that require some level of asymmetry.

### 9.2. Mounting Effects

Mounting from the left side of the horse may meet the lateral needs of both the horse and rider. The typical rider is right-handed and will use their right hand predominately to handle or restrain a horse from the left side. The horse might prefer being approached from the left side due to lateralized sensory and emotional attributes [103]. However, mounting from the ground or from a raised platform using a stirrup causes the saddle to displace toward the mounting (i.e., left) side, and the pressure profile is consistently higher on the right side of the withers [163]. This differential pressure persists even if the rider tries to adjust the position of the saddle. Asymmetries in saddle pressure likely affect nociceptive and biomechanical parameters within affected horses [164,165,166]. There are many other contributing factors that likely impact the horse–rider unit, such as tack fit and use, the environment, ground surfaces, and athletic discipline [6]. It is largely unknown to what degree and in what proportions these factors might contribute to observed left–right differences.

### 9.3. Rein Tension

It seems evident that if a rider wants a horse to travel in a straight line, then equal amounts of rein tension should be applied with both hands; however, symmetric rein tension during riding exercises is very uncommon. Rein tension studies have sought to find relationships between rein tension patterns and rider handedness, which vary both within and between riders [25,167]. A rider’s subjective impression of the rein tension is also reported to not correlate with the actual rein tension [161]. Different riders apply varying amounts of rein tension while riding the same horses through the same dressage test [161]. When riding a symmetrical horse simulator, higher tensions have been reported in the right rein regardless of the rider’s handedness [168]. Rein tension patterns were also not symmetrical on the left and right sides. Asymmetric rein tension is likely influenced by the laterality of both the horse and rider, where the least amount of asymmetry occurs when a right-handed rider is paired with a right-lateralized horse [169].

### 9.4. Saddle Fit

The clinical effects of optimal and improper saddle fit, saddle kinematics, and induced pressures between the horse and saddle have been evaluated [170,171]. The correct saddle fit should enhance the athletic performance of both the horse and rider [172]. An improper saddle fit and use is thought to be a contributory factor to equine back problems, poor attitudes to work, and poor performance [173,174]. However, even with correct saddle fit, asymmetries in the craniocaudal, vertical, and lateral displacement of the saddle have been observed [164,165,166]. Asymmetries in the lateral displacement of the saddle (i.e., saddle slip) have been reported in horses with hind limb lameness [175] and in those judged to be sound [68]. The causes of asymmetric saddle kinematics in horses that are clinically sound remain unknown, but functional asymmetries, sidedness, and motor control issues should be considered.

### 9.5. Rider Influences

Motor laterality in ridden horses is a complex subject since both the horse and rider can be asymmetrical [25]. Handedness in humans is very lateralized, with approximately 90% being right-handed, 9% being left-handed, and 1% being mixed or ambidextrous. It is expected that the effects of handedness in humans directly affect observed left–right differences in any handled or ridden horse. However, it is not clear how strongly linked handedness or other measures of laterality in humans are to the observed left–right differences reported in horses [171]. Rider symmetry, posture, and balance are key indicators of a rider’s skill level [176]; however, rider asymmetries are ubiquitous [63].

Skilled riders can apply appropriate aids via their seat and leg to help rebalance an asymmetric horse [169]. Non-skilled riders may attempt to correct the horse by using the reins, which contributes to asymmetric rein tension. The rider’s hand and leg preferences do not directly confirm that the rider will ride with increased rein tension or adductor strength within the preferred upper or lower limbs. It is often thought that riders who are right-handed will have higher rein tension on the right rein; however, the right rein tension for right-handed riders was reported to have greater variation, and the left rein was more stable and consistent throughout the riding session [33]. This may be explained by the preferred hand or leg being more dexterous when completing tasks, and the contralateral hand is used to provide stability to perform the task. A rider with an asymmetrical position will transfer asymmetrical forces to the saddle and the horse’s back [63,177]. Under the weight of a rider, a horse’s motor laterality also increases [14]. Therefore, it is increasingly difficult to separate left–right differences in the horse from those of the rider [25].

### 9.6. Horse–Saddle–Rider Interactions

Training methods often focus on developing functional symmetry for the horse (e.g., lead changes, turning, and impulsion) [59] and the rider (e.g., balance, strength, and rein tension) [178]. Despite these efforts, many horse–saddle–rider combinations continue to have pronounced left–right structural and functional differences [6]. From a functional perspective, both the horse and rider may have weakness or pain that directly affects their ability to perform as a functional unit [52]. Structurally, scapular asymmetry or muscle atrophy in the horse, a twisted tree or asymmetric saddle flocking, and prior knee injuries and scoliosis within the rider may all have combined detrimental effects [178,179]. The challenge is in determining if the laterality of the horse can be isolated from the tack or the rider’s own laterality [14,169,180].

## 10. Special Sensory Laterality

The special senses of vision, hearing, and olfaction provide important information about the surrounding environment. Sensory laterality is often studied via the response to an external stimulus (e.g., sound or odor) [78,88]. Neuroanatomically, visual and auditory signals are processed predominately within the contralateral cerebral hemisphere, while olfactory stimuli are processed within the ipsilateral cortex [181]. Due to hemispheric specialization, sensory information from one eye or ear would be expected to directly impact measures of laterality in behavioral and locomotor activities [88]. However, limiting afferent signaling to a single eye, ear, or nostril in horses is often difficult and requires well-designed experimental studies [139]. Studies on laterality that attempt to lateralize vision, hearing, and olfaction typically use behavioral or motor responses, which include turning the head, ear movements, and nostril flare (Table 15). However, the authors are not aware of any laterality studies in horses that attempt to assess the sensory component of special senses—visual acuity, electrophysiologic testing (e.g., brainstem auditory evoked responses (BAER)), detection versus response thresholds for smell or taste, equilibrium, or left–right differences in visual, auditory, or olfactory cortical function. It is unknown how predictive observed behavioral or motor responses are for sensory acuity and perception.

### 10.1. Vison

Forward-facing eye placement in humans provides a wide binocular field of view cranially with variable degrees of lateral or peripheral vision. Horses have laterally placed eyes with large monocular fields of view and extensive optic nerve decussation (80–90%) that project afferent sensory fibers primarily to the contralateral hemisphere [185,186,187]. These important anatomical features have been leveraged to assess behavioral and motor responses to visual stimuli [60,139]. However, most studies overlook the effect of temporal and nasal fields within the retina [188] and disregard the 10–20% of optic nerve fibers that do not decussate where a visual stimulus presented to one eye is processed in both cerebral hemispheres [189]. The evaluation of sensory laterality is often complex, as the presentation of visual or auditory stimuli must be carefully controlled [88]. Most studies measure the direction of head movements toward a stimulus and not vision or hearing directly.

Horses are reported to use binocular vision to assess familiar objects and monocular vision to view novel stimuli [60]. Vision laterality has been evaluated by left–right differences in motor responses (i.e., turning toward) or behavioral responses to novel objects (e.g., startle) [60,139] and interactions with other horses and humans [84,91]. However, the axial alignment and positioning of the horse, stimulus, and examiner are of critical importance as a slight left or right deviation may not assess laterality per se, but just the proximity between the horse’s eye, object, or examiner [80]. The lack of an association between a left–right visual bias, different types of stimuli (e.g., novel versus familiar), and hemispheric specialization may be due to an increased reliance on a binocular versus monocular visual field [60] or a lack of motivation or attention in the horse [190]. Laterality is affected not only by the human presence [80] but also by the status of the relationship between the horse and human [147] and the handler’s experience [134,135]. Most studies do not provide a clear description of the experimental settings [186], the specific methods used to limit perspective or human interaction errors, or the consideration of both monocular and binocular visual fields [139].

### 10.2. Hearing

Assessing left–right differences in hearing in humans is routine (i.e., audiology) and relatively easy due to lateral ear placement. However, assessing auditory laterality in horses is more challenging as the ears are closer to the midline and have large, highly mobile pinnae that move independently to detect and localize sounds [181]. Auditory laterality has been assessed by ear movement and head orientation in the direction of auditory stimuli produced by conspecific [19] and human vocalization [87]. Horses tend to first orient their ears towards a sound to localize its source and then turn their heads toward the sound to collect additional visual information [19]. However, the motor response of turning the head toward an auditory stimulus placed either beside or behind the horse does not allow the differentiation of visual versus auditory laterality unless ear movements alone are used to assess auditory laterality. The ear that predominately turns toward or is ipsilateral to the stimulus is judged to be left- or right-lateralized, even though both ears can readily hear and are used to localize sound [181]. There is also a potential anthropomorphic bias in such study designs as humans have ears that are laterally positioned compared to horses.

### 10.3. Olfaction

There are often dramatic differences in the structural and functional features of the olfactory apparatus between species, which needs to be considered in any assessment of olfactory laterality in horses. Olfaction is influenced by nasal structure and breathing patterns [191]. Horses are obligatory nasal breathers, whereas humans and dogs are not. Horses also have very specialized upper airways with a very large nasal cavity and extensive turbinate bones, which change the relative proportions of the nostril, nasal cavity, and nasopharyngeal areas. Behavioral responses to different odors also vary between species (e.g., flehmen response) [192]. Horses use their sense of smell for social interactions and while interacting with their environment (e.g., stallions sniffing feces). Therefore, feces are often used as an olfactory stimulus for horses in a natural setting, while other odors judged to be favorable or noxious from a human perspective are used in experimental settings [18]. The question always arises—does a rose smell like a rose to a horse? Dogs and horses seem to gain valuable information from the prolonged sniffing of feces, which humans cannot or do not want to appreciate. Habituation to odors is well established in humans [193] and appears to also occur in horses [18], which needs to be considered in the design of olfactory lateralization studies.

It is likely that the sense of smell is much more developed in horses than in humans [191,194]. Surprisingly, horses have been reported to be capable of smelling human emotions from sweat samples [183,184]. Humans can verbally describe odors and their responses to them. As there are no specific measures of olfaction in horses, only left–right differences in nostril use [31] and lists of described behaviors that are thought to be associated with olfaction are used to assess laterality [88]. Motor responses to unilateral cotton swab presentation [18] and initial or sustained nostril use have been reported as outcome measures for assessing olfactory lateralization [193]. However, the nostril used or flared could just as easily be the side from which the stimulus was visualized or presented. To add validity to this test, some studies have added measures such as the duration of sniffing, the last nostril used to sniff the object, and recorded non-responders if the horses did not sniff within 2 min after the presentation of a stimulus [18]. Visual cues have not been controlled for or excluded (e.g., unilaterally or bilaterally blindfolded) [1] in most studies that have assessed olfactory laterality in horses [183].

### 10.4. Taste

Lateralization and palatability have also been evaluated by measuring the response to a spectrum of flavors [195]. However, it is not known if horses are able to associate odor and taste and form a concept of flavor like humans [194]. As horses have obligated nasal breathing, the role of olfactory laterality in selecting foodstuffs likely overrides any taste preferences associated with mastication. In addition, vision, olfaction, and hearing each involve afferent signaling via a single cranial nerve lateralized primarily to the ipsilateral (i.e., olfaction) or contralateral (i.e., vision and hearing) cerebral cortex [139]. Taste involves several different types of taste receptors (e.g., sweet, sour, and salt) located within separate regions of the tongue, pharynx, and larynx. Therefore, taste involves several cranial nerves, which greatly increases the complexity of these signaling pathways [196]. Consequently, linking laterality to taste in horses is very challenging, and conclusions should be drawn with extreme caution.

## 11. Dermal Asymmetries

Dermal features such as hair whorls [197] and the pattern or direction of mane placement [81] have been characterized as signs of laterality in horses. Descriptions of directionality for hair whorls include clockwise and counterclockwise rotation and mane positions on or towards a specific side [198]. Hair whorls have been theorized to be caused by lateralized neuronal development [199] and linked to temperament and behavioral characteristics [198]; however, these dermal features have not been linked specifically to hemispheric specialization (Table 16). While there may be obvious left–right differences in the direction of mane positions [81], these dermal characteristics do not meet the definition of laterality. However, it might be possible that there are primary and secondary sources of lateralization. For example, if a horse bends more easily to the right and has an axial skeleton posture or movement pattern that is slightly convex on the right side (i.e., primary source) then the mane might also be more likely to fall on the right side (i.e., secondary source).

## 12. Systematic Perspective

**Figure 4:**
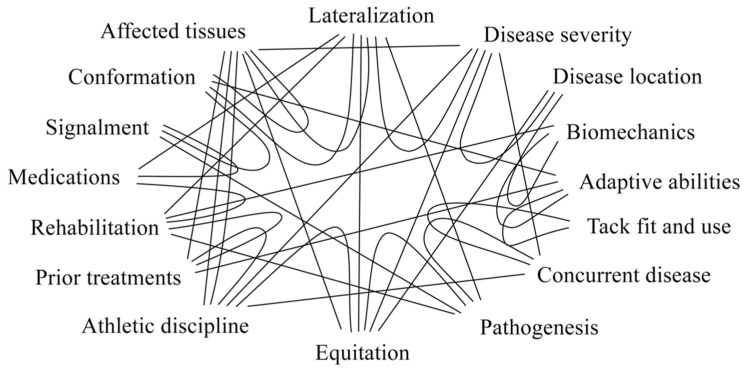
A system-based model illustrating the complex structural and functional interactions contributing to perceived laterality.

Systems biology involves the multidimensional analysis of molecular, cellular, tissue, and organ systems [200]. Due to technological advances in computer modeling, artificial intelligence, and high-throughput measurements, it is now possible to assess the entire genomics, transcriptomics, proteomics, and metabolomics of any item of interest rather than studying a single molecule or an isolated cellular interaction [111]. Using the methodology of systems biology can provide a whole new avenue to assess the multidimensional aspects of sensory, behavioral, and locomotor interactions and the effects of training, human interactions, environmental settings, and treatment (Figure 4). Precision medicine combines systems pharmacology [201] with an individual’s clinical data, digital imaging [202], and biosensor data [203] to generate predictions about disease states and clinical and therapeutic responses [204].

## 13. Managing Laterality and Asymmetries

Most laterality studies proclaim the evolutionary, developmental, or neurophysiologic benefits of being one-sided [9,21,45]. Surprisingly, very few studies have reported negative or detrimental effects [30]. Fewer still include discussions on attempts to change or modify aspects of laterality in horses [1,11]. The questions that arise regarding any type of intervention for modifying or changing the side of laterality include (1) should we change it, (2) can we change it, (3) under what circumstances should we change it (or not), (4) how would we change it, and (5) what are the perceived effects of modifying or changing it? The foundational issue for all these questions is that if laterality is an innate, physiological process due to hemispheric dominance, is any of this even possible? Or are any observed left–right changes, in fact, due to alterations in functional and structural asymmetries and not due to laterality?

### 13.1. Should We Change Laterality?

The basis of most therapeutic approaches in veterinary medicine is to identify a pathological condition and restore the body to a homeostatic or physiological condition. As laterality is considered an innate, physiological process, what basis is there for modifying any observed left–right differences? However, if the observed left–right differences are attributed to functional or structural asymmetries, then it does seem more logical that these processes can or should be modified in some way. If we follow this line of reasoning, then the two primary issues that need to be addressed are as follows: how do we know when an observed left–right difference is (1) due to laterality versus a functional or structural adaptation or (2) a physiological versus a pathological condition? If the observed asymmetries are due to nociceptive or mechanoreceptive defects, then it is our ethical duty to help manage these issues. Chronic pain is deemed to be detrimental to the health and welfare of animals [205]. Altered mechanoreception can also predispose affected animals to altered body awareness and aberrant biomechanics, which increases the risk of tissue trauma and injuries [206]. However, if a horse is using its innate intelligence to navigate life circumstances or compensate for a complex musculoskeletal or neurological condition, the question is, are we, as external observers, intelligent enough to override these deeply interwoven systems? This remains an unanswered question, as interestingly, some horses with significant structural and functional asymmetries are reported to not display a higher rate of lameness [25].

### 13.2. Can We Change Laterality?

Within an individual animal, laterality is often characterized by mixed biases for motor, sensory, and behavioral parameters. Sensory laterality is strongly ingrained and reported to provide advantages in survival due to coordinating cognitive decision processes and rapid fight or flight responses [9]. If visual, olfactory, or auditory afferent signaling could be modified, it is unknown how meaningful changes might be induced. Behavioral responses are affected by temperament, emotions, and social interactions [103,139]. The challenge is knowing if the observed lateralized behavior is innate or learned, rider-induced, or due to pain, fear, or prior trauma [77,80]. Critical periods of development are characterized by windows of time where adaptive behavioral and functional characteristics are generated and strengthened [207]. However, once a critical period has closed, then the associated functional changes are much more difficult to modify [208]. Functional asymmetries due to pain are more readily modified but can often be recurrent.

### 13.3. Circumstances for Changing Laterality?

As structural and functional asymmetries are prevalent and, when extreme, are likely detrimental to the health and welfare of horses, it seems appropriate that minimizing the presence or effects of these asymmetries is warranted. Most training techniques focus on how to correct the horse’s inherent asymmetry with limited or variable results [25]. Diagnostic local anesthesia is used to localize pain, and anti-inflammatory medications are used to provide pain relief and restore symmetric movement patterns. In chronic pain syndromes, compensatory gait mechanisms are common, which are expected to produce additional left–right asymmetries and repetitive use injuries [209,210]. Are there any circumstances where a locomotor bias would be beneficial or appropriate to purposefully induce asymmetry? Racehorses are reported to have a 90% right lead stride pattern unless a forced switch is made while turning or when injured or fatigued [89]. Therefore, it seems reasonable that selecting lateralized behaviors or asymmetries might be needed under certain circumstances to improve performance and racing success.

### 13.4. How Would We Change Laterality?

Structural asymmetries are ubiquitous in horses. The challenge is determining the clinical relevance of subtle asymmetries and selecting what methods, if any, might be used to restore some level of symmetry. Routine hoof care is used to minimize the clinical effects of hoof imbalances. Despite this, many horses continue to have noticeable left–right differences in hoof size, shape, and angulation, which affect limb loading and compensatory limb mechanisms. Many rehabilitation methods are designed to restore postural stability, strength, and flexibility [211]. While left–right differences are evident in many musculoskeletal and neurologic disorders, most treatment approaches restore symmetry in measured outcome parameters at limited amounts or for variable durations. The physiological or conformational sources of structural asymmetries include varying degrees of muscle atrophy, joint angulations, tendon cross-sectional areas, and bone modeling. It can be difficult to know if the larger, taller, steeper, wider, thicker, and longer side is the normal or abnormal side.

### 13.5. What Are the Effects of Changing Laterality?

In humans, there continues to be a seemingly ongoing pursuit of stopping children from using their left hand rather than their right hand to complete tasks [212,213,214]. Attempts to detrain left-handed children often do not produce the desired results for handedness but do create adverse psychological effects [215,216,217]. Similarly, is it better to recognize a horse’s laterality and support their left–right differences [218] or to work against the horse’s innate laterality through extensive training while striving for more symmetrical movements [219]? While the basis of most training techniques is to make horses travel or use their bodies symmetrically, it has been suggested that a rider possibly creates more issues by trying to make a highly lateralized horse more symmetrical [11]. It is likely that different training approaches can be used with the goal of creating greater symmetries versus trying to switch left–right differences to the opposite side. Preventing horses from using a preferred sensory organ by turning their heads away from a stimulus or being led on a certain side may disrupt signal processing within the relevant hemisphere and lead to further stress [1,11]. This becomes critically important when demanding highly skilled movements of the horse–rider unit during riding exercises and competitions [176].

## 14. Conclusions

A plethora of studies have reported left–right differences in horses with the goal of describing behavioral, special sensory, and locomotor effects that are attributed to laterality. However, many observed differences can readily be assigned to functional and structural asymmetries related to pain, human interactions, and training practices. Our biggest challenge is knowing how to differentiate the different types of asymmetries, or in other words, how to recognize true laterality. With an improved understanding of the basic underlying mechanisms of asymmetry and laterality, it is hoped that equestrians, veterinarians, and researchers can better identify and manage issues that are deemed to be detrimental to the health and welfare of horses.

## Figures and Tables

**Table 1:** Terminology and definitions related to laterality.

**Hemispheric specialization**—left–right differences in the neuroanatomic structures of the cerebral cortex or processing of afferent sensory information that produces lateralized behavioral or motor activities.
**Laterality**—observed left–right differences in the function or use of bilateral structures or sides of the body. Synonym: lateralization.

**Table 2:** Terminology and definitions related to bias.

**Bias**—a higher relative proportion of sensory, behavioral, or motor activities localized to either the left or right side of the body.
**Mixed bias**—the proportion of sensory, behavioral, or motor activities that have a nearly equal distribution between the left and right sides of the body.
**Directional bias**—left–right differences in the direction of travel.

**Table 3:** Terminology and definitions related to structural symmetries and asymmetries.

**Structural symmetry**—left–right equivalence in the morphology of paired structures or sides of the body.
**Structural asymmetry**—measurable left–right differences in the morphological features of paired structures or sides of the body.

**Table 4:** Examples of structural asymmetries in horses.

Body Part	Asymmetry
Hoof size and shape	Width and angle differences [44,48,49]
Fetlock joint angle	Joint angle differences [50,51]
Metacarpal bone length	Length differences [44,49]
Dorsal scapula contours	Height differences [44] Lateral prominence differences [52]
Dorsal trunk contours	Left-sided enlargement in wither region [47,53] Epaxial muscle asymmetries [54]
Pelvis	Tuber sacrale height asymmetries due to racing on underbanked curves [55] Tuber coxae height asymmetry due to a pelvic fracture [56]

**Table 5:** Terminology and definitions related to functional symmetry and asymmetries.

**Functional symmetry**—left–right equivalence in the function or use of paired structures or sides of the body.
**Functional asymmetry**—measurable left–right differences in the function or use of paired structures or sides of the body

**Table 6:** Examples of functional asymmetries in horses.

Motor Activity	Signs of Asymmetry
Limb loading	Hooves with lower angles have greater loading [65,66]
Head movement	Asymmetric vertical displacement of the poll in horses with induced forelimb lameness [61]
Wither kinematics	Asymmetric vertical displacement of the withers with different induced head and neck positions [67] Asymmetric vertical displacement of the withers in horses judged to be sound [68,69,70] Asymmetric vertical displacement of the withers in horses with forelimb lameness [62,68,71,72]
Pelvic kinematics	Pelvic movement asymmetries in horses judged to be sound [34] Pelvic movement asymmetries due to induced hind-limb lameness [61]

**Table 7:** Terminology and definitions related to locomotor disorders.

**Soundness**—limb loading and movement patterns that are nearly symmetrical and controlled by physiological processes.
**Gait asymmetries**—minor left–right differences in movement patterns that are thought to be physiological and not clinically relevant. Synonym: altered gait.
**Lameness**—asymmetric limb loading and movement patterns that are judged to be associated with pathological processes.
**Mechanical lameness**—a locomotor disturbance caused by a physical restriction that prevents normal motion of a limb (e.g., fibrotic myopathy).
**Ataxia**—a clinical sign of a neurological disorder characterized by proprioceptive dysfunction, a lack of coordination, or irregular, asymmetric gait patterns.

**Table 8:** The lateralized behavioral and motor responses used to assess emotions, affective states, cognition, and social interactions in horses.

Attribute	Behavioral and Motor Responses
Emotions	Avoidance responses [78] Response to novel stimuli [60,103,139] Agonistic responses to conspecifics [17] Forelimb bias while trailer-loading [40] Response to human approach [80]
Affective status	Social interactions [10] Aggression and reactivity [17] Response to novel stimuli [86] Response to stall confinement [90] Facial expressions [137] Human interpretation of horses’ body language [133]
Cognition	Problem solving [140] Cognitive bias in positive, negative, and ambiguous settings [12] Attention levels across athletic disciplines [73]
Social interactions	Mare–foal interactions [17,141] Affiliative responses to approaches by conspecifics [91] Allogrooming [85] Agonistic responses to conspecifics [10] Auditory responses to conspecific vocalization [19]

**Table 9:** Terminology and definitions related to emotional status.

**Emotions**—a brief duration of intense feelings that are formed in response to an internal or external stimulus or event.
**Mood**—the long-term state of an animal that reflects past experiences and emotional responses. Synonym: affective state.
**Welfare**—the overall emotional and physical state that reflects the balance between positive and negative mental and physical experiences.

**Table 10:** Observed left–right differences in limb position or differences attributed to motor laterality.

Parameter	Attribute
Stance	Relative position of forelimbs [31] Forelimb placement while grazing [27]
Initiation of movement	First fore- or hind limb to move [36,89]
Limb use	Pawing the ground [31,148] Navigating an obstacle [36,149] Stepping up into a trailer [40] Breaking from a starting gate [89]
Kinetics	Unequal limb loading [65,66,124]
Kinematics	Asymmetric vertical displacement of withers [62,67,68]
Locomotion	Leading forelimb at canter or gallop [89] Stride pattern [89] Diagonal dissociation of fore- and hind limbs in trotters [59] Derailment while traveling in a circle [79]

**Table 11:** Terminology and definitions of limb use.

**Mobilizing limb**—the fore or hind limb that is initially moved or more frequently used.
**Stabilizing limb**—the fore or hind limb that is used for support and balance.

**Table 12:** Examples of whole-body asymmetries in horses evaluated at liberty.

Attribute	Signs of Asymmetry
Affiliative approach	Left-sided bias in ponies and riding horses [91] Left-sided bias in feral horses [15] Mixed directional bias in feral horses [10]
Allogrooming	Right-sided bias in feral horses [10] Mixed directional bias in domestic horses [148]
Resting side-by-side	Mixed directional bias in feral horses [10]
Agonistic approach	Left-sided bias in feral horses [17,32] Mixed directional bias in feral horses [10]
Flight response	Left-sided biased for monocular presentation [78] Mixed bias for binocular presentation [78]
Mare–foal interactions	Left-sided biased for foals approaching and traveling with mares [141,158] Left-sided bias for mares traveling with their foals [141]
Suckling	No directional or duration bias in foals [102] No influence of the mare on the suckling side or duration of suckling [102,141]
Side of recumbency	Left-sided bias in foals [141]
Duration of recumbency	Left-sided bias in feral horses [10]
Rolling	No directional bias [36]

**Table 13:** Terminology and definitions related to whole-body movement patterns.

**Sidedness**—left–right differences in the use or ease of movement of the body toward one side compared to the other.
**Straightness**—the ability to adjust the bending of the axial skeleton to match the line of travel equally to the left and right sides.
**Suppleness**—the ability to move easily and equally in opposing directions.

**Table 14:** Examples of whole-body asymmetries in horses evaluated while lunged or during riding exercise.

Attribute	Signs of Asymmetry
Rider-perceived sidedness	Equal suppleness to both sides [37,39] Left bias when lunged or ridden in a circle [95]
Suppleness	Ease of bending in one direction [150]
Straightness	Haunches deviate to one side [150]
Pelvic range of motion	Left bias when walking in a circle [39]

**Table 15:** The behavioral and motor responses used to assess visual, auditory, and olfactory laterality in horses.

Special Sense	Behavioral and Motor Responses
Vision	Mare–foal interactions [141] Spatial relationship to conspecifics [15] Affiliative (positive) responses within a herd [91] Agonistic (negative) responses within a herd [17,32] Avoidance responses [139] Startle and flight responses [78] Emotional responses to objects [60] Breed differences with novel objects [103] Human interactions [84] Responses to human facial expressions [143]
Auditory	Response to familiar versus unfamiliar conspecifics [19] Response to vocally expressed emotions in humans [182] Response to voices from prior human experiences [87]
Olfaction	Response to olfactory stimulus (stallion feces) [31] Response to the spectrum of olfactory stimuli [18] Response to human odors with positive and negative emotions [183,184]

**Table 16:** The behavioral and motor responses attributed to asymmetric dermal features in horses.

Attribute	Behavioral and Motor Responses
Direction of hair whorls	Correlated with temperament and equitation maneuvers [197] Predicts directional bias or ease of movement [198]
Direction of mane placement	Predominantly on the right side in mares [81]

## Data Availability

Not applicable.
